# Empowering Patient Choice: The Essential Need for a Voluntary Advance Directive Framework in Healthcare

**DOI:** 10.1002/alz.083558

**Published:** 2025-01-09

**Authors:** Stephanie Frilling

**Affiliations:** ^1^ LMI, New Freedom, PA USA

## Abstract

**Background:**

**A Medicare Voluntary Advance Directive Framework (Framework)** would enable the creation, storage, and sharing of advance directive documents, ensuring end‐of‐life care appropriately honors the individual and their care wishes, while supporting healthcare teams and family members in making care decisions for their patients and loved ones.

With Medicare enrollment reaching over 65 million beneficiaries in 2023, and Alzheimer’s becoming one of the most expensive conditions ‐ CMS policy makers have a growing responsibility to improve care quality at end‐of‐life.

**Method:**

The federal government will be called upon to assist providers in navigating documents and legal variations of approved forms of directives among the different states, and in ensuring privacy standards that keep an individual’s health data secure. Successful and secure data management at the federal level will be essential to achieving nationwide efficacy in furnishing advance care based upon an individual’s directive.

**Result:**

The lack of a centralized registry for advance directives is particularly felt in underserved and marginalized communities, where access to reliable health care information management is often limited. Our **Framework** addresses this void by offering a dedicated data and document repository. It would serve as a beacon of health equity, meticulously recording and protecting individual health care directives and bridging the availability gap in regions plagued by health disparities. By assuring that advance directives are swiftly accessible and seamlessly incorporated into healthcare delivery, Medicare will improve quality of care and satisfaction for beneficiaries and uphold the dignity of personal health care decisions across all demographics. As a vital element of the health care system, a registry would ensure that all individual’s care preferences are known and honored, facilitating a more consistent and respectful patient experience.

**Conclusion:**

Our poster/presentation identifies challenges associated with managing advanced directives for dementia and Alzheimer’s patients. Federal programs have a responsibility to ensure quality care and appropriate spending. Our work considers public policies and analyze spending for patient who have an advance directive and those that who do not across the health care continuum.

